# Tetrodotoxin: A New Strategy to Treat Visceral Pain?

**DOI:** 10.3390/toxins13070496

**Published:** 2021-07-16

**Authors:** Ana Campos-Ríos, Lola Rueda-Ruzafa, Salvador Herrera-Pérez, Paula Rivas-Ramírez, José Antonio Lamas

**Affiliations:** CINBIO, Laboratory of Neuroscience, University of Vigo, 36310 Vigo, Spain; camposriosana@gmail.com (A.C.-R.); lolarrzg@gmail.com (L.R.-R.); ssalva4@me.com (S.H.-P.); privas84@hotmail.com (P.R.-R.)

**Keywords:** tetrodotoxin, visceral pain, VGSCs

## Abstract

Visceral pain is one of the most common symptoms associated with functional gastrointestinal (GI) disorders. Although the origin of these symptoms has not been clearly defined, the implication of both the central and peripheral nervous systems in visceral hypersensitivity is well established. The role of several pathways in visceral nociception has been explored, as well as the influence of specific receptors on afferent neurons, such as voltage-gated sodium channels (VGSCs). VGSCs initiate action potentials and dysfunction of these channels has recently been associated with painful GI conditions. Current treatments for visceral pain generally involve opioid based drugs, which are associated with important side-effects and a loss of effectiveness or tolerance. Hence, efforts have been intensified to find new, more effective and longer-lasting therapies. The implication of VGSCs in visceral hypersensitivity has drawn attention to tetrodotoxin (TTX), a relatively selective sodium channel blocker, as a possible and promising molecule to treat visceral pain and related diseases. As such, here we will review the latest information regarding this toxin that is relevant to the treatment of visceral pain and the possible advantages that it may offer relative to other treatments, alone or in combination.

## 1. Introduction

Visceral pain is a complex common symptom in patients with gastrointestinal (GI) conditions. Although pain is normally an adaptive response warning of impeding danger, anomalous pain originating from internal organs may be the result of aberrant signaling in which visceral afferents become hypersensitive. This response usually results in hyperalgesia (an increase in the sensitivity to painful stimuli) and allodynia (abnormal painful sensations provoked by non-painful stimuli) [1], impeding patients from leading a normal life. As such, visceral pain represents a considerable healthcare burden.

The visceral hypersensitivity commonly originates at diverse locations, making its detection and definition complicated. It may be the result of an insult that causes acute pain, possibly due to inflammation in a visceral organ (e.g., pancreatitis), flow occlusion (e.g., kidney stones), nerve injury driving neuropathic pain, a functional visceral disorder (e.g., Irritable Bowel Syndrome—IBS) or even cancer [2,3,4,5,6]. Moreover, chronic pain may arise from altered peripheral or central nociception without any evidence of physical damage, resulting in functional pain that was recently described as nociplastic pain [3,7]. The sensitive hyperresponsiveness is thought to arise through the activation of nociceptive afferent fibers by noxious stimuli, or as a result of a nerve damage that alters nerve conduction (e.g., spontaneous firing) or that modifies neurotransmitter properties [1,8]. A promising approach to the treatment of visceral pain is the blockade of ion channels in the peripheral primary afferents that drive painful stimuli [9]. There is evidence of a relationship between voltage-gated sodium channels (VGSCs) and pain transduction. Significantly, these channels are key proteins in neuronal excitability and sensory transduction, as they are responsible for the depolarization of the resting membrane potential and the subsequent initiation of the action potential (AP) in neurons [10,11].

One of the greatest challenges in pain treatment is the identification of the true source of pain. However, the World Health Organization’s (WHO’s) analgesic ladder establishes different strategies to treat pain regardless of its origin. As such, non-steroid analgesics are proposed for the treatment of mild pain, while antiepileptic drugs are usually employed for more intense pain and opioids are used to treat severe pain. Moreover, as pain is not easy to assess, these drugs may be combined with adjuvants depending on the patient. Despite the advances in understanding the pathophysiology and pharmacology of GI conditions, there are still many limitations to their successful treatment [12]. A few drugs specifically ameliorate visceral pain [13], although they are commonly associated with adverse side-effects or poor effectiveness [5,11,14,15]. Furthermore, patients treated with opioid drugs often develop tolerance to their prolonged use. Hence, there remains a clear need to develop novel therapies to treat visceral pain, which have focused the spotlight on ion channels as potential therapeutic targets. Although Transient Receptor Potential (TRP) channels were the first ion channels to be associated with pain sensations, especially the TRP Vanilloid 1 (TRPV1) channel that is activated by capsaicin [16], VGSCs have also been attributed a key role in pain transduction. In fact, these ion channels are already targeted by drugs approved to treat mild pain, such as lidocaine, and their contribution to pain transduction make them a promising therapeutic target.

Tetrodotoxin (TTX) is a lethal neurotoxin produced by bacteria such as *Shewanella putrefaciens*, and it is a selective sodium Na^+^ channel blocker that has generated considerable interest in recent years. TTX is ingested by pufferfish, different marine bivalves and gastropods, and the trophic chain is thought to be the main source of its accumulation [17]. Before the toxin had been identified, fugu fish were used in traditional Japanese medicine to cure neuralgia in leprosy patients. Subsequently, TTX was discovered, extracted and purified and used to dampen spasms in patients with tetanus [18]. This toxin dramatically dampens the firing of APs by specifically blocking the VGSC pore, thereby disrupting the passage of Na^+^ [11,18,19]. As such, TTX has been proposed as a possible strategy to manage pain by blocking VGSCs. Hence, here we shall discuss the most relevant studies into the use of TTX to manage visceral pain.

## 2. Primary Sensory Innervation of the Viscera

**Figure 1:**
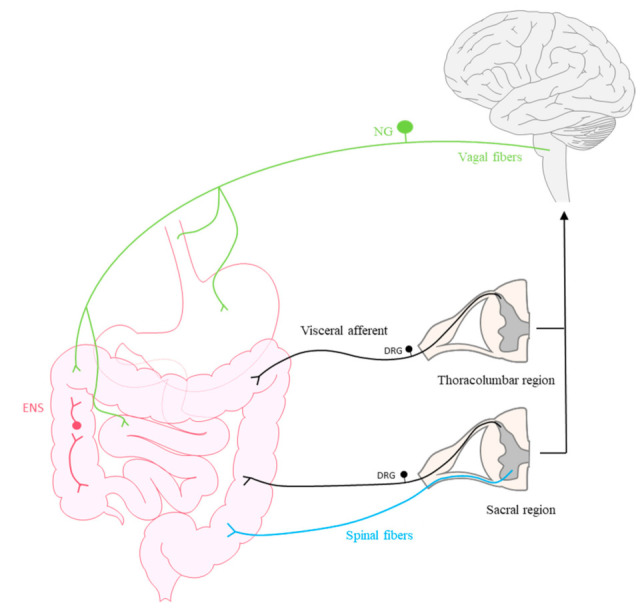
Scheme representing the primary sensory innervation of the gastrointestinal tract. The vagus nerve (green) has its cell bodies in the nodose ganglion (NG). Extrinsic primary afferent neurons (visceral afferent, in black) located in thoracolumbar and sacral dorsal root ganglion (DRG) and spinal fibers innervate the viscera and drive stimuli to the central nervous system. Furthermore, an intrinsic nervous system, the enteric nervous system (ENS, red) locally innervates the gastrointestinal tract.

VGSCs are widely distributed throughout the central and peripheral nervous systems (CNS and PNS, respectively), and they are expressed by sensory afferents innervating viscera. Two main types of nervous afferents are implicated in transmitting visceral pain stimuli to the CNS (see Figure 1).

The first are extrinsic primary afferent neurons (EPANs) located in thoracolumbar and sacral dorsal root ganglia (DRG) whose axons reach the dorsal horn, from where neurons project to numerous brain regions related to visceral pain responses [12,20,21,22,23]. The second are sensory afferents that drive stimuli through the vagus nerve via neurons located in the nodose ganglion (NG) and pelvic nerves, which, despite innervating the same visceral organs, project to different regions [21,23,24]. It is thought that by activating regions of the brainstem, vagal fibers might also fulfil a modulatory role in terms of descending pain sensation [3]. Furthermore, a special feature of visceral innervation is that in addition to extrinsic innervation, most organs have intrinsic primary afferent neurons (IPANs) that contribute to the enteric nervous system (ENS). The cell bodies of these neurons reside within the organs themselves and they are primarily dedicated to physiological functions such as GI motility, secretion or blood flow [3,5,12,25,26]. The ENS is comprised of two plexuses of neurons, and it extends from the esophagus to the anus. The first neural network is known as the myenteric plexus (Auerbach’s plexus) and it controls motility, while the second is the submucosal plexus (Meissner’s plexus) that controls secretory activities [26]. Accordingly, a bidirectional relationship exists between the intestine and the CNS, referred to as the gut–brain axis, which involves: the autonomic nervous system, both sympathetic and parasympathetic; the circulatory system, in which different neurotransmitters participate such as serotonin, catecholamines, dopamine or short chain fatty acids (SCFAs); the hypothalamic–pituitary–adrenal (HPA) axis; and the immune system [27,28]. Furthermore, intestinal dysbiosis, an imbalance in the composition of the intestinal microbiota has been associated with many GI alterations, including painful visceral disorders such as IBS [29].

As mentioned above, visceral pain is characterized in refer to the body wall, where afferent somatosensory neurons in the DRG are key controllers of this referred pain. This way, pain can be considered a convergence of afferent visceral and somatic sensory pathways in the spinal cord and higher regulatory centers [30,31,32,33,34]. It is worth mentioning that lamina I is the first site in the spinal cord where somatic and visceral C-fibers directly converge onto an individual projection, integrating the complex causes of referred pain. Indeed, it is thought that lamina I contains different groups of neurons, which provide somatic, visceral and somatovisceral inputs to the CNS [33,35].

## 3. VGSCs

Primary sensory neurons that detect noxious stimuli in visceral organs are commonly pseudounipolar neurons with polymodal terminals, and they express a wide range of ion channels and G-protein coupled receptors (GPCRs) that transduce different painful sensations [3,10]. VGSCs are expressed by visceral sensory neurons and they are the main contributors driving noxious stimuli from the PNS to the CNS [36]. Indeed, several studies have shown an association between VGSCs and visceral pain-related pathologies [37,38,39,40,41,42]. Significantly, these ion channels play a fundamental role in C-type fiber activity and in sensory perception [37,43], and mutations in this family affect the normal function of the viscera in animal models and humans [36,44,45,46,47,48].

The channels of the VGSC family are formed by one α-subunit that forms the ion conduction pore and one or two β-subunits that modulate the channel’s activity. VGSCs are classified into nine subfamilies based on their α-subunit (Nav1.1–Nav1.9), encoded by distinct SCN genes. Each α-subunit contains four domains connected by three intracellular loops and each domain is formed by six transmembrane segments, the voltage-sensitive properties of VGSCs being dictated by the fourth transmembrane segment of each domain [14,49,50]. VGSC channels contain six toxin binding sites and TTX binds specifically to toxin site 1 that resides in the first transmembrane segment. When TTX is attached to the channel it physically occludes the pore, impeding Na^+^ ions from passing through the channel [51].

Na^+^ channels play a key role in the generation and propagation of APs, making them crucial elements in neurotransmitter release from sensory nerve terminals [10]. They drive depolarization throughout the neuronal membrane and when this depolarization reaches the axon terminal, calcium channels open and the exocytosis of synaptic vesicles is provoked, driving neurotransmitter release. This neurotransmitter release differs depending on the location and distribution of the Na^+^ channels, such that the latter determines a neuron’s functional properties [52]. For example, Nav1.3 is expressed strongly by human and mouse enterochromaffin (EC) cells, and its activation is involved in the excitability in these cells, modulating serotonin release [53]. Serotonin is implicated in the pathophysiology of IBS since alterations in its signaling could affect motor and secretory activities, inducing IBS [54]. It was also found that the local activation of Na^+^ channels through specific activators such as Tityustoxin (TsTX) modulates glutamate release, increasing calcium influx in some specific brain regions [52,55]. Moreover, Nav1.7 is required for neurotransmitter release in the olfactory glomerulus [56]. Thus, TTX will disrupt neurotransmitter liberation by binding to site 1 of toxin sensitive VGSCs, dampening the activity of VGSCs related to pain pathways.

Many studies have found different VGSCs to be widely distributed throughout the CNS and PNS [13,51,53,57]. Among the array of Na^+^ channels, Nav1.1, Nav1.6, Nav1.7, Nav1.8 and Nav1.9 are those most strongly linked to pain pathways. Nav1.2, Nav.1.3 and Nav1.6 are expressed by myenteric neurons [58], while elsewhere, Nav1.3 and Nav1.7 were seen to be the only TTX-sensitive (TTX-S) Na^+^ channels expressed by these neurons [59]. Furthermore, Nav1.2 and Nav1.9 are expressed by submucosal neurons, and Nav1.7 in the DRG innervates the intestine and it is expressed in sympathetic and sensory fibers [14,58,60]. The nodose and jugular ganglia of the vagus nerve express Nav1.7, Nav1.8 and Nav1.9, and, to a lesser extent, Nav1.6 [61]. Furthermore, functional Nav1.8 expression has been found in the superior cervical ganglion of the sympathetic nervous system. Hence, TTX-S and TTX-resistant (TTX-R) Na^+^ channels expressed in the autonomic nervous system (ANS) and the ENS might play a fundamental role in driving visceral pain stimuli.

Classically, TTX has been used as a selective and reversible Na^+^ channel blocker to characterize VGSCs [11,62] based on their sensitivity, whereby TTX-S are blocked by nanomolar concentrations of TTX and TTX-R channels require higher micromolar concentrations to be inactivated [14,63]. Interestingly, it has long been known that damage to sensitive peripheral nerve fibers promotes the expression of TTX-S channels, which might explain the overexcitability associated with pain [64,65,66]. The identification of VGSCs as candidates for visceral hypersensitivity has drawn attention to TTX as a potential analgesic drug for visceral pain. The efficacy of TTX depends on the differential expression of VGSC subtypes in excitable fibers of different tissues, and many studies have implicated TTX-S channels in normal and pathological visceral pain modulation.

### 3.1. TTX-S Channels

As mentioned above, the pain-related TTX-S channels Nav1.1–Nav1.4, Nav1.6 and Nav1.7 are distributed widely throughout the CNS and PNS [36]. Mutations in these channels underlie several nervous system disorders, such as seizures (Nav1.2) [67], paralysis and myotonia (Nav1.4) [68] or pain transmission disorders (Nav1.1, Nav1.3, Nav1.6 and Nav1.7) [69,70,71]. Here, we will focus on the main TTX-S channels related to visceral pain (as summarized in Table 1).

Nav1.1 is expressed broadly in the PNS and it has been mostly related with mechanical pain perception [78]. The pain response caused by δ-theraphotoxin-Hm1a venom administered by intraplantar injections is mediated by the TTX-S Nav1.1 in the DRG and trigeminal ganglion, and it is blocked by TTX (0.5 µM) [79]. Nav1.1 was also found to be functionally expressed in mechanosensory colonic fibers [79]. Elsewhere, an increase in Nav1.1 channel expression was detected in chronic visceral hypersensitivity (CVH) disorders, and the inhibition of these channels in mechanical IBS models of pain relieved suffering by decreasing the Na^+^ currents in colon-innervating DRG neurons [69]. As CVH is an extended GI problem in IBS patients, these data suggest it would be interesting to study whether the blockade of Nav1.1 with TTX might relieve mechanical pain associated with IBS. It is also worthwhile to consider the use of TTX to ease mechanical pain associated with GI related problems, especially those that cause visceral hypersensitivity and abdominal referred visceral pain.

Nav1.6 is encoded by the *SCN8A* gene, and it is closely related to mechanical pain transmission and pain sensations in the lower viscera. Specifically, this channel appears in the endings of stretch-sensitive colorectal unmyelinated sensory afferents of the pelvic nerve and it is activated by colorectal distension. Indeed, colorectal nociception can be attenuated by targeting Nav1.6 with TTX and by other specific modulators, such as μ-conotoxin GIIIa, μ-conotoxin PIIIa [70] and β-scorpion toxin Cn2 [74]. Thus, it would be of interest to consider the use of TTX to treat colon and rectal pain, targeting pelvic afferents.

Nav1.7 is expressed in the DRG, the sympathetic ganglia, the myenteric and trigeminal ganglia and NG neurons, specifically in primary nociceptive neurons [80,81,82]. This is the main TTX-S channel implicated in the conduction of APs in Ah and C-fibers of the vagal sensory system [83], and it participates in the initiation of APs in sensory neurons in response to depolarization driven by noxious stimuli [44,84,85]. Many studies have shown Nav1.7 to be essential for pain transduction, including inflammation-associated pain, heat- and cold-related pain and mechanical nociception, and TTX has been proven to be a very effective analgesic in treating burn-associated [86], cancer-related [87] or neuropathic pain [88].

It is also thought that mutations in the *SCN9A* gene would affect voltage-dependent gating, such that Nav1.7 channels would open more readily or they would be less affected by inactivation, thereby leading to overstimulation and generating sensations of pain [45]. Several pain disorders are related to Nav1.7 channel dysfunction. For example, the gain-of-function mutations in this gene have been linked with paroxysmal extreme pain disorder [75] and erythermalgia [45]. Altered levels of this channel were also found in rectal sensory fibers of patients with rectal hypersensitivity [89]. A Nav1.7 knockout mice has been developed that shows no painful behaviors [44,45,90,91], while elsewhere, rare loss-of-function mutations in the α-subunit of this gene that are strongly expressed in nociceptive neurons are also associated with an inability to sense pain [44,45,90,91].

In terms of visceral pain, the value of Nav1.7 as a visceral pain target is not clear. Recent studies showed that mutations in the *SCN9A* gene are implicated in IBS, together with interleukin 2 (IL-2), an immune mediator altered in IBS. This suggests that Nav1.7 is involved in the neuro-immune mechanisms associated with visceral pain and that TTX might effectively regulate CVH in IBS patients [89]. It was proposed that a combination of Nav1.7 and TRPV1 inhibitors might be effective in these circumstances, combating the overexpression of these channels found in extreme paroxysmal pain disorder patients with rectal hypersensitivity [89]. In a study of the peritoneum of women with endometriosis, TRPV1 and SCN9A (Nav1.7) mRNA expression was enhanced [42], although other recent studies demonstrated that Nav1.7, despite being related to acute and somatic pain, is not required for visceral pain signaling. In visceral models of pain, TTX reversed pain responses in a Nav1.7 conditional knockout mouse treated with mustard oil and capsaicin, indicating that this channel does not appear to be crucial in sensory neurons expressing nociceptive markers [92]. Indeed, a conditional nociceptor specific Nav1.7 knockout mouse did not differ in its pain response from wild-type mice following intracolon instillation of capsaicin and mustard oil or in a cyclophosphamide-induced pain model of cystitis [93]. The deletion or antagonism of Nav1.7 does not interfere with the noxious mechanical and chemical stimuli or in the blockade of mechanosensitivity induced by TTX (100 μM). Hence, while there is a clear dependency on TTX-S currents in visceral afferents, these are not driven by Nav1.7.

Despite the loss of interest in inhibiting Nav1.7 with TTX to target visceral pain, this drug may still be of interest to manage referred visceral pain. In fact, μ-TRTX-Hhn1b is a 35 amino acid peptide in *Ornithoctonus hainana* tarantula venom that acts as a selective Nav1.7 inhibitor, and it has been shown to be effective in relieving abdominal constriction-related hyperalgesia by inhibiting Nav1.7 and blocking the pain transduction [91,94]. This suggests that the use of TTX might also be similarly effective.

All this information reveals that TTX-S channels are probably more closely implicated in pain that was at first thought, and the TTX could represent an important analgesic resource for pain treatment.

### 3.2. TTX-R Channels

TTX-R Na^+^ currents are driven through Nav1.5, Nav1.8 and Nav1.9, and they are more strongly expressed in PNS sensory neurons than TTX-S channels. Nevertheless, these channels also contribute to neuronal excitability, even though their axonal expression is not sufficient to support AP conduction [95]. It has been seen that morphine tolerance and persistent visceral inflammation results in visceral hyperalgesia through changes in the TTX-R Na^+^ channels in rats [40], and enhanced TTX-R channel expression in DRG and NG neurons was detected during gastric inflammation and gastric hyperalgesia [39,96]. Similarly, exposure to TTX (10 µM) completely inhibited AP propagation in both A and C fibers from the isolated vagus nerve [95].

The TTX-R Nav1.5 channel is expressed in the ENS, spinal visceral afferents and Cajal interstitial cells, and it is thought to be mostly responsible for GI motility [38,63,97]. Various studies have found that loss-of-function mutations in *SCN5A*, the gene that encodes the Nav1.5 channel, are behind IBS symptomatology [38,48,72]. Moreover, several channelopathies are related to mutations in *SCN5A* gene, such as Brugada syndrome (a common heritable syndrome that affects cardiac rhythm) or cardiac fibrillation and dilated cardiomyopathy [46,98,99]. Curiously, long QT syndrome, a channelopathy also associated with mutations in this gene, has been related to GI symptoms and patients with this condition also experience intestinal problems [73]. Thus, the relationship between Nav1.5 and GI pathologies should be further studied to identify strategies that combine the use of TTX and other blockers and that successfully attenuate visceral hypersensitivity in IBS patients.

Although it was thought that TTX-R Na^+^ channels are not relevant to AP conduction, Nav1.8 was found to be a major contributor to the upstroke of APs in sensory neurons. Indeed, Nav1.8 is essential for cold pain perception [100] and it exerts a significant influence on neuropathic pain [101]. This channel was first recognized as a sensory neuron specific channel, and classically, it has been related to general pain [37]. It is expressed strongly by NG neurons that project nociceptive C-fibers to the lungs and by DRG neurons [82]. Mutations in the *SCN10A* gene that codes for Nav1.8 are associated with a chronic mouse model of infectious jejunitis, and they fulfil a crucial role in the appearance of prolonged sensory neuron hyperexcitability in peritoneal somatosensory DRG neurons [76]. Moreover, Nav1.8 mRNA upregulation is implicated in bone cancer pain [94,102]. Nav1.8 null mice experience weak pain in response to intracolonic mustard oil instillation and weaker signs of referred hyperalgesia, suggesting a role of this channel in mediating spontaneous nociceptor activity. However, a painful behavioral response of Nav1.8 null mice is evident in a model of cystitis and referred hyperalgesia, similar to that of wild-type mice, indicating that Nav1.8 is not required for the visceral transduction of noxious chemical stimuli [103]. As TTX reverses pain behavior in a model of induced cystitis [76], the transduction of this kind of noxious stimuli might be driven through TTX-S currents and hence, TTX might represent an important analgesic treatment.

Nav1.9 is encoded by the *SCN11A* gene, and it is expressed strongly by vagal sensory afferents in the NG and DRG. It has also been found in intrinsic myenteric neurons [63,82,104]. Similar to the other VGSCs, Nav1.9 participates in the modulation of APs, reducing the AP threshold. Mutations in the *SCN11A* gene are involved in chronic inflammatory pain, diabetic neuropathy and orofacial neuropathic pain [77], and as Nav1.8, it participates in bone cancer pain [102].

## 4. VGSC Blockade by TTX as a New Pain Therapy

The most common visceral pain suffered by individuals is related to GI processes, although GI pain pathophysiology remains an enigma. This condition is a worldwide problem and it is considered a recurrent problem in terms of seeking medical attention. IBS is one of the most prevalent forms of GI pain, affecting nearly 10–15% of the population in Europe and the United States [105,106]. The lack of suitable treatments affects the management of this type of pain and there is a clear need for more intense research to find effective visceral pain therapies. The non-selective VGSC blockers used to treat visceral pain are notably much more advanced and they have been seen to be broadly effective for a wide range of pain-related applications, although they have many side effects. Moreover, classical therapies with the VGSC blocker lidocaine still carry many risks when employed over the long-term [63], making this counterproductive when treating chronic afflictions. In terms of visceral pain, TTX has proven to be an effective analgesic, not only in experimental protocols [92,93,107] but also in clinical studies into cancer-related pain [87] (see Table 2). Indeed, it has shown promising effectiveness when used in animal models of different types of visceral conditions.

**Figure 2:**
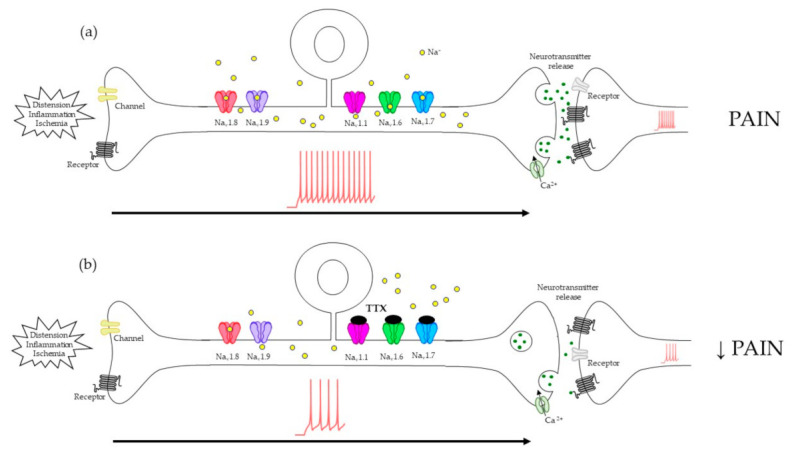
Mechanism proposed for the blockade of tetrodotoxin (TTX) sensitive voltage-gated sodium channels (VGSCs) to diminish pain transduction in primary sensory neurons. (**a**) After a noxious stimulus reaches the depolarization threshold, TTX-sensitive and -resistant VGSCs are activated, generating action potentials (APs) along the axon to the presynaptic terminal. Here, neurotransmitter release activates the postsynaptic neuron, and the stimulus is transmitted to the central nervous system, evoking pain sensations. (**b**) By blocking TTX-sensitive VGSCs with TTX, AP transmission is dampened and consequently, painful sensations decrease.

In a schematic model of TTX action as an analgesic drug (Figure 2), low doses will only be effective against TTX-S channels. However, this toxin might not only work as a therapy itself when TTX-R channels are involved but also as a co-adjuvant along with other VGSC blockers or classic pain drugs that block TTX-R channels or reduce chronic visceral pain. Furthermore, its application along with other treatments could improve its penetration trough biological barriers [108]. This would drastically limit the current problems of adverse side-effects, low effectiveness or the tolerance developed through prolonged opioid-analgesic use.

Recent clinical trials investigating the use of TTX on cancer-related pain has shown clear advantages in the management of chronic conditions through TTX-mediated VGSC blockade. The application of twice daily subcutaneous injections of TTX for 4 days caused a strong analgesic effect against cancer-related visceral pain in patients [87,109,110,111]. Moreover, the analgesic effect of subcutaneous TTX administration has been tested in visceral-specific animal models of pain [92]: hyperalgesia caused by intracolonic administration of capsaicin and mustard oil and cystitis bladder pain induced by cyclophosphamide. The subcutaneous administration of TTX significantly reduced both visceral pain behavior and referred hyperalgesia through VGSCs, yet without eliminating pain completely. It should be noted that TTX acts in a dose-dependent manner in all cases and it did not cause any locomotor side-effects at the highest dose (6 µg/kg) [92]. Other studies on a visceral mouse model of pain showed that TTX (doses of 3 and 6 µg/kg) and morphine produced significant antinociceptive effects by diminishing abdominal contractions, possibly through Nav1.7 inhibition [11], the morphine causing marked sedation, and no side-effects were attributed to TTX use [11].

Further experimental evidence has demonstrated the effects of TTX as a pain killer in animal models by blocking Nav1.7 [94], as well as Nav1.6 channels [70]. Both these channels are expressed strongly on processes of sensory neurons innervating colorectal tissue, and a colorectal distension model of pain showed that they are more strongly activated and that they trigger colorectal pain. Indeed, application of TTX on the mucosal and serosal side of colorectal tissue dampens the pain response by predominantly blocking Nav1.6 [70]. Moreover, TTX has proven to be a very effective analgesic in treating burn-associated [86] and neuropathic pain [88]. Elsewhere, it was shown to also have a crucial role in relieving vaginal pain in endometriosis and vulvodynia, common problems in women of reproductive age that generate prevalent visceral pain [42,57].

Regarding Nav1.5, Nav1.8 and Nav1.9 channels, the use of TTX as a therapeutic agent is complicated by the high concentration required to block TTX-R VGSCs. Thus, it would be desirable to find strategies that combine TTX with other blockers to successfully attenuate visceral hypersensitivity. For example, Nav1.8 anti-sense treatment demonstrated that these channels are involved in afferent nerve sensitization after chemical irritation of the rat bladder, suggesting they represent a new target to treat visceral inflammatory pain [112]. Furthermore, Nav1.5 is related to GI pathologies in IBS patients and in cardiac syndromes. Here, we find another barrier to the therapeutic use of TTX, since the heart is one of the very few organs that is not affected by TTX, even at high and lethal doses [113]. Thus, again it is necessary to define strategies that combine TTX and other blockers that successfully attenuate visceral hypersensitivity.

A remarkable problem when treating visceral hypersensitivity such as GI pain is the lack of selectivity for different VGSC subtypes. Many molecules are being studied in an attempt to find selective blockers of Nav1.7, such as ProTx-II (a spider venom peptide) [114], AMG8379 [115], PF-05089771 [116], XEN402 [117], BIIB074 [118], GpTx-1, PF-04856264 and CNV1014802 (raxatrigine) [119]. Furthermore, ICA-121431 selectively blocks the Nav1.1 and Nav1.3 channel subtypes [79], and ambroxol and A-803467 are being studied to treat neuropathic pain through Nav1.8 blockade [120,121].

Due to the dose-response effects of TTX, it is very important to control the concentrations of this drug used in clinical trials and in experiments on animal models. Unless the limitations of its analgesic use are related to its toxic effects, some studies have focused on the safest doses that can be applied to relieve pain. The LD_50_ (50% of its lethal dose) of TTX in mice is 10.7 μg/kg for intraperitoneal administration, 12.5 μg/kg for subcutaneous administration and 532 μg/kg for intragastric administration. In rabbits, the minimal lethal dose (MLD) is 5.3 μg/kg for intramuscular injection and 3.1 μg/kg for intravenous injection [122]. It was also found that TTX is 50 times less lethal via oral administration than via intraperitoneal injection [122], although this would not be the most effective way to treat patients without producing undesirable side-effects. In humans, it was concluded that 30 µg twice twice daily for 4 days produces analgesia with well-tolerated side-effects [87,110], also without producing genotoxic effects [123]. Hence, the medical application of TTX might not imply undesirable toxic effects at adequate doses, which means that it could be used in future treatments to combat visceral pain.

## 5. Concluding Remarks

In the light of all these data, we can conclude that both TTX-S and TTX-R channels are implicated in the development of visceral pain. One promising analgesic strategy would be the blockade of TTX-S VGSCs with TTX in primary sensory neurons, which would effectively diminish pain transduction, even if TTX-R channels were also expressed there (as illustrated in Figure 2). Although selectivity and systemic toxicity of TTX constrains its clinical use and considering that TTX-S channels are not the only VGSCs that drive painful stimuli, TTX represents a promising scaffold to develop more specific inhibitors. Furthermore, combinations of TTX with other selective blockers of TTX-R VGSCs might be a suitable strategy to efficiently palliate visceral pain.

In conclusion, TTX is a promising analgesic with potential to treat visceral pain and especially, painful GI conditions. New research might enhance the effectiveness of TTX related therapies to treat visceral pain.

## Figures and Tables

**Table 1:** Main tetrodotoxin-sensitive and tetrodotoxin-resistant voltage-gated sodium channels (VGSCs) related to visceral pain.

VGSCs	Gene	Tetrodotoxin	Associated Pain	Alteration	References
Nav1.1	*SCN1A*	Sensitive	Irritable bowel syndrome	Upregulation	[61]
Nav1.5	*SCN5A*	Resistant	Irritable bowel syndrome, cardiac syndrome	Loss of function	[38,48,72,73]
Nav1.6	*SCN8A*	Sensitive	Colorectal pain	Activation	[74]
Nav1.7	*SCN9A*	Sensitive	Erythromelalgia, paroxysmal extreme pain	Gain of function or altered functional levels	[45,71,75]
Nav1.8	*SCN10A*	Resistant	Chronic jejunitis pain	Change in function or expression	[76]
Nav1.9	*SCN11A*	Resistant	Chronic inflammatory pain	Potentiation	[77]

**Table 2:** Clinical and experimental evidence of the use of TTX to manage visceral pain.

	Model	Administration of Tetrodotoxin (TTX)	Outcome	References
**Clinical evidence**	Cancer-related pain patients	Subcutaneous injection (30 µg)	Long-lasting analgesia (56.7 days)	[87,109]
**Experimental evidence**	Wild-type and Nav1.7 knock-out mouse models of pain	Subcutaneous injection (0.1, 0.3, 1, 3, 6 µM)	- TTX reduced both visceral pain-behaviour and referred hyperalgesia - Dose-dependent actions - Antinociceptive effect	[11,92]
	Dorsal root ganglion (DRG) neurons	Perfusion (0.5 µM)	TTX diminished Nav1.1 activated by Hm1a	[79]
	Colorectal distension model of pain	Mucosal and serosal application (1, 3, 10 µM)	- 10 μM TTX on the mucosal side and 1 µM on the serosal side attenuated afferent responses to stretch - TTX predominantly inhibited Nav1.6	[70]
	Colorectal afferents sensitized by IL-2	Perfusion (1 µM)	TTX blocked Nav1.7	[89]
	Chemical sensitized afferent splanchnic fibers	Perfusion (0.1 µM)	- TTX blocked pain-related behaviour - TTX failed to inhibit Nav1.7	[93]
	Vagus nerve of rats	Perfussion (10 µM)	TTX blocked action potential propagation	[95]
	Pelvic vaginal afferents	Perfusion (0.5 µM)	TTX significantly reduced vaginal afferent responses to mechanical stimuli	[57]
	Vagina-innervating DRG neurons	Perfusion (0.1 µM)	TTX decreased neuronal excitability by blocking TTX-S channels	
	Vaginal pain mouse model	Intravaginal administration (0.5 µM)	TTX reduced spinal cord neuronal activation	
	Bladder afferents of mice	Instillation (1 µM)	TTX supresed mechanical distension and reduced firing	[107]
	Bladder-innervating DRG neurons	Incubation (100 nM)	TTX reduced Na^+^ current density and excitability	
	In vivo bladder of mice	Intravesical infusion (1 µM)	TTX reduced noxious bladder distension-induced nociceptive signalling	

## Data Availability

Not applicable.
